# Determining the risk factors of malaria and anemia in children between 6 and 59 months using the joint generalized linear mixed model on the 2021 Nigeria Malaria Indicator Survey dataset

**DOI:** 10.3389/fpubh.2024.1503884

**Published:** 2025-01-06

**Authors:** Talani Mhelembe, Shaun Ramroop, Faustin Habyarimana

**Affiliations:** School of Mathematics, Statistics, and Computer Science, University of Kwazulu-Natal, Pietermaritzburg, South Africa

**Keywords:** anemia, malaria, children, Nigeria, joint modeling

## Abstract

**Background:**

Malaria and anemia are significant public health concerns that contribute to child mortality in African. Despite global efforts to control the two diseases, their prevalence in high-risk regions like Nigeria remains high. Understanding socioeconomic, demographic, and geographical factors associated with malaria and anemia, is critical for effective intervention strategies.

**Objective:**

The study aims to assess the risk factors for malaria and anemia in children under 5 years using the joint generalized linear mixed model (JGLMM).

**Methods:**

The Nigeria Malaria Indicator Survey (NMIS) 2021 dataset was used, with a sample of 10,120 children aged 6–59 months. A two-stage sampling method was applied. Descriptive statistics and chi-square tests examined factors associated with malaria and anemia prevalence. Missing data were handled using multiple imputations with chained equations (MICE). Lastly, the adjusted odds ratio was interpreted for the current study. SAS was used in analyzing the data and statistical significance was set at 5% significance level.

**Results:**

The prevalence of malaria and anemia was 36.81 and 67.66%, respectively, in children between 6 and 59 months old in Nigeria. The JGLMM was used to examine malaria RDT and anemia findings in conjunction with demographic, geographic, and socioeconomic covariates. The following underlying risk factors for malaria and anemia in children were discovered in the study: region, altitude, age of child in months, toilet facility of the household, main wall material used for the house, main roof material used for the house, children under five who slept under a mosquito net, whether the child had fever in last 2 weeks before the survey took place, place of residence where the child resides, household wealth index, sex of child, and mother’s education level. However, whether the mother knew of ways to prevent malaria was not statistically significant regarding anemia.

**Conclusion:**

This study highlights the importance of addressing fever as a key factor for anemia and improving housing conditions to reduce malaria and anemia prevalence. Policymakers should prioritize mosquito net distribution and healthcare access, particularly in rural areas. The study’s novelty lies in its handling of missing data through imputation techniques, enhancing the reliability of findings.

## Introduction

Anemia in children under 5 years of age, defined by the World Health Organization (WHO) as a hemoglobin concentration less than 11 g/dL, is regarded as a global public health problem (1). Children under five assume a disproportionate burden of anemia, defined by the World Health Organization as a hemoglobin (Hb) level of less than 11 g/dL, and is an increasingly prevalent global health problem (1). Although it is estimated that almost half of the anemia cases are due to iron deficiency (2), the other causes of anemia, which also disproportionately affect children and pregnant women, are multifactorial and include nutritional deficiencies and parasitic infections (3, 4).

Despite all efforts made to curb anemia, it continues to be one of the critical public health problems affecting children globally in both developing and developed countries. Based on the report from the World Health Organization (WHO) (1), it is one of the 10 most serious health problems globally. Approximately about 273 million children between 6 and 59 months old across the world were suffering from anemia in 2011, according to a WHO report, with prevalence rate of 42.4%. Furthermore, the estimated prevalence of anemia in children between 6 and 59 months old is 62.3% in Sub-Saharan Africa (SSA) (roughly about 85 million). The 2018 Nigeria Demographic Health Survey (NDHS) data has revealed that the prevalence of anemia among children between 6 and 59 months old was also high, anemia affected about 68% of the children; with 38% having moderate anemia, 27% mild anemia, and 3% having severe anemia.

Despite the success of malaria reduction in Nigeria, some areas still have a high prevalence of malaria parasitemia (5). Malaria is a significant contributor to anemia, with severe anemia (a hemoglobin level below 8 g/dL), as the main manifestation of complicated malaria. Both conditions are known to contribute to the considerable burden of morbidity and mortality, especially among children under 5 years of age (5, 6). According to the National Malaria Control Programme (NMCP), malaria alone has been shown to contribute to between 30 to 50% of outpatient visits (7), 15–20% of hospital admissions, and 20% of hospital deaths, with most of this burden borne by children under 5 years and pregnant women (6). Anemia alone also affects over 50% of individuals in the same population (6), making both illnesses a great public health concern. The causative role of malaria, particularly Plasmodium falciparum, in anemia is particularly important in malaria-endemic regions, including much of sub-Saharan Africa (SSA) (3). Given the etiologic role of malaria in anemia and the recent scale-up of malaria prevention and control activities, the prevalence of anemia, if other factors responsible for anemia remained stable, would be expected to decrease (3).

The parasites that cause malaria are transmitted to humans through the bite of the female *Anopheles* mosquito, and it takes 10–15 days to develop symptoms of the disease after being infected (1, 8). Malaria is more transmitted during the high temperature and rainy seasons. Malaria is not contagious. However, it is possible to contract the disease from another person through blood transfusions or organ transplants (1, 9). Several interventions and precautions are taken against malaria, but the disease remains a major health problem globally, especially in developing countries (1). Twenty-nine countries accounted for 96% of malaria cases globally, and four countries, Nigeria (27%), the Democratic Republic of the Congo (12%), Uganda (5%), and Mozambique (4%), accounted for almost half of all the cases globally (1). The WHO African Region, with an estimated 234 million cases in 2021, accounted for about 95% of all cases (10). Globally, the malaria mortality rate halved from 30 in 2000 to 15 in 2015; it then continued to decrease but at a slower rate, falling to 14 in 2019. In 2020, the mortality rate increased to 15.1 before slightly decreasing to 14.8 in 2021 (1).

In order to apply successful implementation to reduce the burden of malaria substantially, there is a continuous need to understand the epidemiology and risk factors associated with the disease (4, 11). Many studies have identified various risk factors associated with malaria infection (12), including socioeconomic, environmental, demographic, and others (4, 9, 13).

Based on the high prevalence of malaria and anemia in children from Nigeria, it was worthwhile to investigate what are some of the risk factors that contributes to the high prevalence for both diseases.

The main objective of the current study is to determine the malaria and anemia risk factors among children under 5 years of age from Nigeria. The method that will be used to achieve the objective is the joint generalized linear mixed model (JGLMM), that will be used to model data from the demographic health surveys; particularly the Nigeria Malaria Indicator Survey (NIMS) 2021.

## Methodology and material

### Study area

This study uses the 2021 Nigeria Malaria Indicator Survey (NMIS), which was implemented by the National Malaria Elimination Programme (NMEP) of the Federal Ministry of Health (FMoH) in collaboration with the National Population Commission (NPC) and the National Bureau of Statistics (NBS) (7). A two-stage sampling strategy was adopted for the 2021 NMIS.

The 2021 NIMS sample was stratified and selected in two stages. Stratification was achieved by separating the 36 states and Federal Capital Territory into urban and rural areas. There were 73 sampling strata since there are no rural areas in Lagos. Samples were selected independently in every stratum through a two-stage selection. Implicit stratification was achieved at the lower administrative levels by sorting the sampling frame before sample selection according to administrative order and using probability proportional to size selection in the first sampling stage.

In the first stage, 568 enumeration areas (EAs) were selected with probability proportional to the EA size. The EA size is the number of households residing in the EA. The sample selection was done in such a way that it was representative of each state. The result was 568 clusters throughout the country, 195 in urban areas, and 373 in rural areas. In the second stage’s selection, a fixed number of 25 households were selected in every cluster through equal probability systematic sampling.

Three questionnaires were used in the 2021 NMIS: the Household Questionnaire, the Woman’s Questionnaire, and the Biomarker Questionnaire (7). Based on The DHS Program’s model questionnaires, the questionnaires were adapted to reflect the population and health issues relevant to Nigeria. After the questionnaires were finalized in English, they were translated into Hausa, Yoruba, and Igbo (7). The National Health Research Ethics Committee of Nigeria (NHREC) and the ICF Institutional Review Board reviewed and approved the survey protocol (7).

Using the DHS data from the 2021 NIMS, the children’s sample was chosen using the household recode. The data collection took place from October to December 2021. The children’s total population was 12,180; according to the NIMS report, the selected children for anemia and RDT testing were between 6 and 59 months old (7). Therefore, after applying the filter only to children between 6–59 months old, the sample used in this study became 10,120 (7). There were some missing observations for some of the predictors, and a complete case analysis was performed. In the current study the power was calculated to investigate if the sample selected will give adequate power to detect the true effect of exposures. The following was done to determine the power:

- Step 1: found the total number of eligible children from the Central (1920), North (4879), and South (3321) regions.
- Step2: calculated the mean and standard deviation, from the three regions and found it to be 3,374 and 1480.179, respectively.
- Step 3: Proc Power in SAS studio was used to calculate the power, where the one sample means was adopted.

The computed power was found to be >0.999, which implies that the sample size used in this study has an extremely high probability of detecting a statistically significant effect, implying that parameter estimates are accurate.

Multiple imputation by chained equations (MICE) was used to deal with the missing values, the outcome of the imputation was then used in modeling the data by Joint Generalized Linear Mixed Models (JGLMM). The section below outlines the imputation methods that can be applied to missing data.

In the current study, children who were under 5 years old were selected for analysis purposes. Furthermore, those whose caregivers gave consent for hemoglobin levels to be measured from the children were selected, and those who tested for malaria using rapid diagnostic testing (RDT) were selected.

### Missing data

It is usual to encounter missing data in epidemiological studies (14–16). Most survey data contain missing observations (17), and different methods can be employed to handle missing observations (15, 17). The technique employed can affect the analysis outcome (15). This could compromise the conclusion drawn from the results (18). In most cases, missing data is managed by dropping the cases that are not fully measured (15). However, this study compared the complete case and imputed data based on statistics of the standard error resulting from the JGLMM joint model. The comparison has been thoroughly documented in the results section.

The assumption was that the data was missing at random (MAR) (17). When multiple imputation is applied to a, that is, the MAR dataset and unbiased results with accurate estimates for the standard error (SE) (19). In this study, multiple imputation by chained equations was used. Chained equations are also known as fully conditional specification (FCS) (15), and it is the algorithm adopted by SAS Enterprise. This approach is more flexible to imputation since it is designed to handle different types of variables (binary, continuous, categorical, and ordinal) and does not assume the multivariate normality of the data (15, 20). Assuming the multivariate normality can often lead to truncation and inflated standard errors or wrongful inference with the loss of information (15). As applied in SAS, imputation by FCS is also an iterative process that starts by imputing every missing value with random draws from the distribution of the non-missing values (15). In this study, the FCS has been employed for imputation using two iterations, with several imputations (nimpute = 4).

### Variables

The current study considered two response variables. The first one was malaria status for children under the age of 5 years. The status of malaria was determined using RDT to check if the child had malaria (positive) or not (negative). The second one is anemia status in children, which is determined based on the hemoglobin concentration level in the blood measured in grams per deciliter (g/dl) (9, 13). When the hemoglobin concentration level adjusted for altitude is less than 11 g/dL, the child is considered anemic, otherwise not anemic (13, 21).

### Independent variables

The independent variables used in this study were also used in previous literature and involved several demographic, geographic, and socio-economic factors (3, 4, 6, 8, 9, 22–24). The current study used independent variables assumed to be associated with anemia and malaria, such as region, altitude, child’s age in months, toilet facility, main floor material, whether the children slept under a mosquito net or not, whether the child had fever in the last 2 weeks or not, type of place of residence, household wealth index, sex of child, electricity, mother’s education level, main wall material, main roof material, and whether the parent knows about ways to prevent malaria. In Nigeria, the studies that modeled anemia and malaria also used the same independent variables found in the current study (5, 8). However, some variables were assumed to be associated with malaria and or anemia in some parts of Africa (4, 9, 13). The independent variables not included in their studies are whether the household has electricity, whether children under 5 slept under a net, main roof material, main wall material, and altitude level.

### Statistical analysis

The current method used is Statistical Software Suite (SAS) to clean the data. In addition, the bivariate method was analyzed in SAS, and cross-tabulation techniques were applied. Pearson’s chi-square test and *p*-values were used to investigate whether the independent variables selected for this study were associated with each one of the response variables or not. The frequencies and percentages were used to summarize the data, and the *p*-values were used to check the relationship between the independent and response variables. Based on the chi-squared test, all the independent variables were statistically significant, with a *p*-value less than 0.05. The analysis of multivariate used the SAS University edition (online) PROC GLIMMIX procedure. The procedure enabled us to jointly model two outcomes (response) variables with similar distributions, link functions, or different link functions (13). However, this study found a similar distribution and link functions for the two outcome variables (13). Furthermore, all the possible interactions were assessed, and none of them were significant (13, 25).

### Rationale over selecting the GLMM

GLMMs are extensions of Generalized Linear Models (GLMs) that include both fixed effects (for population-level effects) and random effects (to account for variability across different clusters, or individuals). They are versatile and can handle different types of distributions for the outcome variable, such as binary outcomes, count data, or continuous data. GLMMs are commonly used for binary dependent variables by applying a logit link function (or log-odds) to model the probability of an event occurring (in this case either having malaria or being anemic).

In summary, GLMMs are fully applicable to binary outcomes like malaria and anemia, and they allow for accountability for both fixed effects (predictors) and random effects. In this case the assumption is that the two outcomes are related, therefore the use of joint modeling was necessary.

### Model formulation

The current study considered two response variables: anemia and malaria status of a child under five. Suppose that the response variable $x_{j1}$ is anemia status; anemic is assigned to a child with anemia, and not anemic is assigned to a child who does not have anemia. The second response variable as $x_{j2}$ to be malaria RDT status, where one (1) is assigned as positive status and zero (negative) status. The distinguished results emerge from the bivariate Bernoulli distribution, with $q_{j1}$ as the likelihood of anemia occurring in child j and $q_{j2}$ as the probability of malaria occurring in child j (13). Therefore, the binary generalized linear model can be written as:


(1) $$h_{1}\left(\pi_{j1}\right)=Y_{j1}\theta_{1}+U_{j1}w_{1}$$

(2) $$h_{2}\left(\pi_{j2}\right)=Y_{j2}\theta_{2}+U_{j2}w_{2}$$

Where $\theta_{1}$ and $\theta_{2}$ are assumed to be the vectors of fixed effects, $w_{1}$ , and $w_{2}$ are the vectors of the random effects, while $Y_{j1},Y_{j2},U_{j1},\mathrm{and}\;U_{j2}$ are the designed matrices for fixed effects and random effects, respectively (13, 25). Therefore, the equation of the variance–covariance matrices model is shown as follows:


(3) $$w=\left(\begin{array}{c} w_{1}\\ w_{2} \end{array}\right)\sim i.i.d.MVN\left(0,\sum \right)=MVN\left(\left[\begin{array}{c} 0\\ 0 \end{array}\right],\left[\begin{array}{cc} \Sigma_{11} & \Sigma_{12}\\ \Sigma_{21} & \Sigma_{22} \end{array}\right]\right)$$

Where the $\Sigma_{11}$ and $\Sigma_{22}$ , are the variance components of anemia of children under 5 years and malaria respectively, while $\Sigma_{12}$ and $\Sigma_{21}$ are the correlation components between anemia and malaria are the same (13, 25). When the correlation components from Equation 3, $\Sigma_{12}$ = $\Sigma_{21}$ = 0, the multivariate joint model under the generalized linear mixed model becomes a separate model (13, 25). Equation 4 is the joint generalized linear mixed model of the malaria and anemia status of each under 5 years’ children:


(4) $$\begin{array}{l} \left(\begin{array}{c} Malaria\\ Anemia \end{array}\right)=\\ \left(\begin{array}{c} b_{11}Region\\ b_{21}Region \end{array}\;\begin{array}{c} +b_{12}Altitude+\ldots +b_{113}PreventionKnowledge\\ +b_{22}Altitude+\ldots +b_{212}Mother^{\prime }sEducationLevel \end{array}\right)\\ +\left(\begin{array}{c} \varepsilon_{1}\\ \varepsilon_{2} \end{array}\right) \end{array}$$

## Results and interpretation

### Comparison of the complete case and imputed data

Results from the three different analyses show that, in general, the results are similar. The results from the current study are similar to the study by Hendry et al. (15). A comparison of the results of the complete case analysis (CC) with the other analysis (imputation) shows that the standard errors (SE) of the estimated coefficients for the CC are larger in all the predictor variables. There is little difference in the magnitude when the estimated coefficients are compared. Based on the results presented in Table 1, the SE found for the data where imputation has been employed is lower; therefore, it shows higher precision in the estimates found when using the imputed data. In the current study, the estimates were made using the 10 iterations of the FCS method in SAS. As the number of iterations increases, the number of combinations tested for the imputed data also increases, and this gives a larger range of degrees of freedom (15). This indicates that the estimates have stabilized and are trusted (15).

**Table 1:** Estimated coefficients and standard errors for the predictors selected in the different analysis.

Category	Malaria						Anemia					
	Complete case		Five iterations		Ten iterations		Complete case		Five iterations		Ten Iterations	
	Estimate	Standard error	Estimate	Standard error	Estimate	Standard error	Estimate	Standard error	Estimate	Standard error	Estimate	Standard error
Region (ref = South)												
Central	−2.9179	0.2559	−2.788	0.1263	−2.744	0.1261	–	–	0.6278	0.1721	0.6278	0.1721
North	−2.6633	0.2573	−2.5484	0.127	−2.5043	0.1268	0.914	0.351	0.9102	0.1736	0.9102	0.1736
Altitude (ref = Greater than 1,000 m)												
0–500 m	0.7699	0.2112	0.5716	0.07202	0.22	0.074	0.661	0.1654	0.6898	0.08448	0.6686	0.08266
501–1000 m	0.5623	0.2241	0.5227	0.07582	0.5677	0.1119	0.39	0.1789	0.4062	0.09132	0.3935	0.08933
Age in months (ref = 6–12)												
12–24	0.5393	0.06744	0.5215	0.03333	0.5215	0.03334	−0.309	0.09864	−0.318	0.0491	−0.318	0.0491
25–36	0.8162	0.06749	0.8035	0.03333	0.8031	0.03333	−0.79	0.09711	−0.8055	0.0483	−0.8055	0.0483
37–48	0.9558	0.06672	0.9285	0.03297	0.928	0.03298	−1.074	0.09547	−1.0937	0.0475	−1.0937	0.0475
49–59	1.185	0.06669	1.1632	0.03293	1.1631	0.03293	−1.14	0.09538	−1.1596	0.04741	−1.1596	0.04741
Toilet Facility (ref = Toilet with Flush)												
Latrine	0.174	0.0524	0.1584	0.02597	0.1585	0.02598	0.245	0.07058	0.2554	0.03505	0.2554	0.03505
No Facility	0.3011	0.05705	0.2878	0.02823	0.2873	0.02822	0.209	0.07894	0.2312	0.0391	0.2312	0.0391
Main Wall Material (ref = Wood/Mud)												
Bricks	−0.4337	0.1183	−0.442	0.05873	−0.4478	0.05867	–	–	−0.2666	0.08357	−0.2666	0.08357
Cement	−0.3496	0.04985	−0.3575	0.02472	−0.3526	0.02472	−0.25	0.0758	−0.2464	0.03764	−0.2464	0.03764
No walls	−0.6132	0.09439	−0.5857	0.04675	−0.5948	0.04674	–	–	−0.2117	0.07121	−0.2117	0.07121
Other	−0.6636	0.142	−0.6551	0.0698	−0.643	0.06936	–	–	–	–	–	–
Main roof material (ref = Wood)												
Asbestos			0.2098	0.07962	0.2299	0.0793	–	–	–	–	–	–
Metal/Zinc	0.1627	0.06759	0.1546	0.03344	0.153	0.03345	–	–	–	–	–	–
Other			−0.2312	0.08043	−0.2273	0.08001	–	–	–	–	–	–
Thatch/palm leaf	−0.355	0.07782	−0.3677	0.03849	−0.3608	0.03852	–	–	−0.2558	0.06003	−0.2558	0.06003
Slept under mosquito net (ref = Some)												
All	–	–	−0.1199	0.02476	−0.1216	0.02477	–	–	−0.08391	0.03703	−0.08391	0.03703
No	–	–	−0.08427	0.02447	−0.08626	0.02448	–	–	–	–	–	–
Had fever (Ref = Yes)												
Do not know	−0.6167	0.3145	−0.6265	0.1572	−0.6253	0.1572	−0.861	0.4197	−0.8703	0.2099	−0.8703	0.2099
No	−0.5301	0.03315	−0.5327	0.01642	−0.5332	0.01642	−0.386	0.04858	−0.3833	0.02409	−0.3833	0.02409
Place of residence (ref = Urban)												
Rural	0.2541	0.04157	0.2706	0.02053	0.2714	0.02053	–	–	0.1238	0.02701	0.1238	0.02701
Wealth Index (ref = Richest)												
Middle	0.8351	0.08804	0.7763	0.04329	0.7814	0.04332	0.254	0.1088	0.2462	0.0538	0.2462	0.0538
Poorer	1.0641	0.1047	1.0037	0.0516	1.0121	0.05161	0.408	0.138	0.3854	0.06835	0.3854	0.06835
Poorest	1.2331	0.116	1.1556	0.05715	1.1624	0.05715	0.568	0.1589	0.5304	0.07858	0.5304	0.07858
Richer	0.7725	0.07206	0.7302	0.03539	0.7312	0.03542	0.226	0.08192	0.2251	0.04057	0.2251	0.04057
Sex of child (ref = Male)												
Female	−0.08627	0.03188	−0.08559	0.01579	−0.08593	0.01579	−0.131	0.04485	−0.1305	0.02224	−0.1305	0.02224
Education Level (ref = Secondary)												
Higher	–	–	−0.1297	0.03754	−0.1301	0.03755	−0.3	0.08089	−0.3136	0.04007	−0.3136	0.04007
No education	0.5217	0.04873	0.5211	0.02411	0.5207	0.02411	0.246	0.06961	0.2387	0.03452	0.2387	0.03452
Primary	0.4152	0.05146	0.414	0.0255	0.4127	0.02551	0.182	0.07301	0.1829	0.03626	0.1829	0.03626
Are there ways to prevent malaria (ref = Do not know)												
Yes	–	–	−0.1263	0.03226	−0.1263	0.03226	–	–	–	–	–	–

### Univariate results

The results from Table 2 indicate the frequency distribution and percentages of childhood anemia and malaria, respectively, with the independent variables. Cross-tabulation techniques were used to analyze the data and summarize the results in both Tables. Pearson’s chi-square test and the *p*-values were used to investigate whether the independent variables were statistically significant to the response variables. Based on the outcomes of Table 2, all the independent variables are associated with childhood anemia and malaria, with a *p*-value less than 0.05.

**Table 2:** Distribution of the frequency of the predictor variables.

Variables	Category	Anemia status		*p-*value	Malaria status		*p*-value
		Anemic	Not Anemic		Negative	Positive	
Altitude level	0 to 500 m	6,063 (59.2%)	2,823 (57.56%)	<0.0001	5,535 (48.1%)	3,542 (30.8%)	<0.0001
	501 to 1,000 m	787 (7.68%)	401 (3.92%)		1,309 (11.4%)	1,063(9.2%)	
	Greater than 1,000 m	80 (0.78%)	88 (0.86%)		41(0.3%)	10(0.1%)	
Age in months	6 to 12	1764 (17.22%)	534 (5.21%)	<0.0001	766 (7.48%)	220 (2.15%)	<0.0001
	12 to 24	1,488 (14.53%)	707 (6.90%)		1,564 (15.27%)	734 (7.16%)	
	25 to 36	1,453 (14.19%)	915 (8.93%)		1,378 (13.45%)	817 (7.97%)	
	37 to 48	1,425 (13.91%)	971 (9.48%)		1,427 (13.93%)	942 (9.19%)	
	49 to 59	800 (7.81%)	185 (1.81%)		1,339 (13.07%)	1,058 (10.33%)	
Region	Central	1,166 (11.38%)	754 (7.36%)	<0.0001	1,221 (10.6%)	528 (4.6%)	<0.0001
	North	3,594 (35.09%)	1,273 (12.43%)		3,705 (32.2%)	3,509 (30.5%)	
	South	2,170 (21.19%)	1,285 (12.55%)		1959 (17.0%)	578 (5.0%)	
Toilet facility	Latrine	3,235 (31.81%)	1,151 (11.32%)	<0.0001	3,126 (27.4%)	2,916 (25.5%)	<0.0001
	No facility	1781 (17.51%)	714 (7.02%)		1,146 (10.0%)	1,074 (9.4%)	
	Toilet with flush	1872 (18.41%)	1,416 (13.92%)		2,560 (22.4%)	608 (5.3%)	
Main wall material	Bricks	126 (1.24%)	65 (0.64%)	<0.0001	102 (0.9%)	53 (0.5%)	<0.0001
	Cement	3,597 (35.45%)	2,253 (22.2%)		4,092 (35.8%)	1,631 (14.3%)	
	No walls	211 (2.08%)	81 (0.80%)		135 (1.2%)	112 (1.0%)	
	Other	110 (1.08%)	40 (0.39%)		39.4 (0.3%)	13 (0.1%)	
	Wood/Mud	2,830 (27.89%)	835 (8.23%)		2,454 (21.5%)	2,788 (24.4%)	
Main floor material	Cement	2,780 (27.38%)	1,600 (15.76%)	<0.0001	2,939 (25.8%)	1,302 (11.4%)	<0.0001
	Ceramic tiles	618 (6.09%)	492 (4.85%)		958 (8.4%)	159 (1.4%)	
	Earth/Sand	3,254 (32.05%)	1,051 (10.35%)		2,752 (24.1%)	3,059 (26.8%)	
	Other	142 (1.40%)	100 (0.99%)		129 (1.1%)	38 (0.3%)	
	Wood	83 (0.82%)	32 (0.32%)		44 (0.4%)	26 (0.2%)	
Main roof material	Asbestos	87 (0.86%)	66 (0.65%)	<0.0001	178 (1.6%)	36 (0.3%)	<0.0001
	Cement	77 (0.76%)	44 (0.43%)		101 (0.9%)	10 (0.1%)	
	Metal/Zinc	5,435 (53.59%)	2,688 (26.51%)		5,307 (46.6%)	3,489 (30.6%)	
	Other	87 (0.86%)	28 (0.28%)		71 (0.6%)	37 (0.3%)	
	Thatch/Palm leaf	672 (6.63%)	267 (2.63%)		430 (3.8%)	400 (3.5%)	
	Wood	513 (5.06%)	177 (1.75%)		718 (6.3%)	624 (5.5%)	
Slept under mosquito net	All	2,427 (23.75%)	1,099 (10.75%)	<0.0001	2,426 (21.1%)	1778 (15.5%)	<0.0001
	No	3,511 (34.36%)	1823 (17.84%)		3,479 (30.3%)	1905 (16.6%)	
	Some	980 (9.59%)	379 (3.71%)		965 (8.4%)	929 (8.1%)	
Had fever	Do not know	14 (0.14%)	11 (0.11%)	<0.0001	18 (0.2%)	8.10 (0.1%)	<0.0001
	No	4,061 (39.65%)	2,355 (22.99%)		4,546 (39.5%)	2,426 (21.1%)	
	Yes	2,855 (27.88%)	946 (9.24%)		2,321 (20.2%)	2,181 (19.0%)	
Place of residence	Rural	5,100 (49.79%)	2,109 (20.59%)	<0.0001	4,611 (40.1%)	3,939 (34.3)	<0.0001
	Urban	1830 (17.87%)	1,203 (11.75%)		2,273 (19.8%)	675 (5.9%)	
Wealth index	Middle	1,428 (13.94%)	678 (6.62%)	<0.0001	1,343 (11.7%)	780 (6.8%)	<0.0001
	Poorer	1,489 (14.54%)	499 (4.87%)		1,237 (10.8%)	1,432 (12.4%)	
	Poorest	1,610 (15.72%)	457 (4.46%)		1,281 (11.1%)	1,647 (14.3%)	
	Richer	1,356 (13.24%)	772 (7.54%)		1,332 (11.6%)	533 (4.6%)	
	Richest	1,047 (10.22%)	906 (8.85%)		1,693 (14.7%)	223 (1.9%)	
Sex of child	Female	3,290 (32.12%)	1,663 (16.24%)	0.0095	3,401 (29.6%)	2,111 (18.4%)	<0.0001
	Male	3,640 (35.54%)	1,649 (16.10%)		3,482 (30.3%)	2,503 (21.8%)	
Electricity	No	4,327 (42.55%)	1,640 (16.13%)	<0.0001	3,472 (30.4%)	3,534 (30.9%)	<0.0001
	Yes	2,561 (25.18%)	1,641 (16.14%)		3,359 (29.4%)	1,064 (9.3%)	
Education level	Higher	516 (5.04%)	511 (4.99%)	<0.0001	886 (7.7%)	154 (1.3%)	<0.0001
	No Education	3,167 (30.92%)	1,036 (10.12%)		2,582 (22.5%)	2,879 (25.0%)	
	Primary	1,046 (10.21%)	446 (4.35%)		1,070 (9.3%)	826 (7.2%)	
	Secondary	2,201 (21.49%)	1,319 (12.88%)		2,347 (20.4%)	756 (6.6%)	
Knowledge of preventative measures	No	980 (9.57%)	414 (4.04%)	0.0258	748 (7.30%)	647 (6.32%)	<0.0001
	Yes	5,498 (53.68%)	2,703 (26.39%)		5,355 (52.27%)	2,848 (27.80%)	
	Do not know	452 (4.41%)	195 (1.90%)		371 (3.62%)	276 (2.69%)	

The current study’s prevalence of malaria and anemia was 36.81 and 67.66%, respectively. Table 2 shows that the results from the study show that the prevalence of anemia was higher in children from a household that is at an altitude less than 500 meters (59.2%) and lower in those from a household that is at an altitude between 501 to 1,000 meters (7.68%), and an altitude that is greater than 1,000 meters (0.78%). The same results also revealed that as the age of the child increases, the prevalence of anemia decreases; the prevalence is as follows: (17.22%), (14.53%), (14.19%), (13.91%), and (7.81%), for 6 to 12, 13 to 24, 25 to 36, 37 to 48, and 49 to 59 months old, respectively. The prevalence of anemia was higher in children from rural areas (49.7%) compared to (17.87%) of those who live in urban areas. Furthermore, the prevalence of anemia was higher in children from the poorest household (15.72%), (14.54%) from poorer, middle (13.94%), richer (13.24%), and richest (10.22%). The study further showed that the prevalence of anemia is higher in children whose mother has no education (30.92%), secondary education (21.49%), primary education (10.21%), and higher education (5.04%).

The results from Table 2 show the prevalence of malaria in children from an area where the altitude is between 0 and 500 meters (30.8%), 501 to 1,000 meters (9.2%), and an altitude of greater than 1,000 meters (0.1%), respectively. The prevalence of malaria in children who are between 49–59 months old is (11.6%), compared to children who are between 37–48, 25–36, and 12–24 months, with a prevalence of (10.1%), (9.6%), and (8.9%), respectively. Children from rural areas showed a high prevalence of malaria at (34.3%) compared to those from urban areas with a (5.9%) prevalence. Children from a household where the wealth index is in the poorest class showed a high prevalence of malaria at (14.3%), followed by those from the poorer, middle, richer, and richest at (12.4%), (6.8%), (4.6%), and (1.9%), respectively. Furthermore, children whose mother has no education showed a high prevalence of malaria at (25.0%), compared to those children whose mother has primary, secondary, and higher education at (7.2%), (6.6%), and (1.3%), respectively.

### Multivariate results

The multivariate analysis was used in SAS PROC GLIMMIX to assess the correlation between malaria, anemia, and the selected independent variables associated with the two diseases. All possible interactions between the independent variables were checked, and none were statistically significant. Therefore, they were not included in the model results presented in Table 3.

**Table 3:** Statistical analysis model results on malaria and anemia determinants.

Category	Malaria				Anemia			
	Estimate	Standard error	*p*-value	Odds ratio	Estimate	Standard error	*p*-value	Odds ratio
Region (ref = South)								
Central	−2.744	0.1261	<0.0001	0.064312581	0.6278	0.1721	0.0003	1.873484376
North	−2.5043	0.1268	<0.0001	0.081732791	0.9102	0.1736	<0.0001	2.484819448
Altitude (ref = Greater than 1,000 m)								
0–500 m	0.22	0.074	0.003	1.246076731	0.6686	0.08266	<0.0001	1.951503303
501–1000 m	0.5677	0.1119	<0.0001	1.764204711	0.3935	0.08933	<0.0001	1.482159284
Age in months (ref = 6–12)								
12–24	0.5215	0.03334	<0.0001	1.684552584	−0.318	0.0491	<0.0001	0.727602788
25–36	0.8031	0.03333	<0.0001	2.23245081	−0.8055	0.0483	<0.0001	0.446864438
37–48	0.928	0.03298	<0.0001	2.529445225	−1.0937	0.0475	<0.0001	0.334974791
49–59	1.1631	0.03293	<0.0001	3.199837413	−1.1596	0.04741	<0.0001	0.3136116
Toilet Facility (ref = Toilet with Flush)								
Latrine	0.1585	0.02598	<0.0001	1.171751924	0.2554	0.03505	<0.0001	1.290977909
No Facility	0.2873	0.02822	<0.0001	1.332824001	0.2312	0.0391	<0.0001	1.260111236
Main Wall Material (ref = Wood/Mud)								
Bricks	−0.4478	0.05867	<0.0001	0.639032478	−0.2666	0.08357	0.0014	0.765979402
Cement	−0.3526	0.02472	<0.0001	0.70285828	−0.2464	0.03764	<0.0001	0.781609519
No walls	−0.5948	0.04674	<0.0001	0.551672889	−0.2117	0.07121	0.003	0.809207423
Other	−0.643	0.06936	<0.0001	0.525712917	–	–	–	–
Main roof material (ref = Wood)								
Asbestos	0.2299	0.0793	0.0037	1.258474156	–	–	–	–
Metal/Zinc	0.153	0.03345	<0.0001	1.165324979	–	–	–	–
Other	−0.2273	0.08001	0.0045	0.796681742	–	–	–	–
Thatch/palm leaf	−0.3608	0.03852	<0.0001	0.697118408	−0.2558	0.06003	<0.0001	0.774296813
Slept under mosquito net (ref = Some)								
All	−0.1216	0.02477	<0.0001	0.885502499	−0.08391	0.03703	0.0235	0.919514009
No	−0.08626	0.02448	0.0004	0.917355688	–	–	–	–
Had fever (Ref = No)								
Do not know	−0.6253	0.1572	<0.0001	0.535100874	−0.8703	0.2099	<0.0001	0.418825883
No	−0.5332	0.01642	<0.0001	0.586724444	−0.3833	0.02409	<0.0001	0.681608386
Place of residence (ref = Urban)								
Rural	0.2714	0.02053	<0.0001	1.311799685	0.1238	0.02701	<0.0001	1.13178949
Wealth Index (ref = Richest)								
Middle	0.7814	0.04332	<0.0001	2.184528466	0.2462	0.0538	<0.0001	1.279155379
Poorer	1.0121	0.05161	<0.0001	2.751372835	0.3854	0.06835	<0.0001	1.470202285
Poorest	1.1624	0.05715	<0.0001	3.19759831	0.5304	0.07858	<0.0001	1.699612017
Richer	0.7312	0.03542	<0.0001	2.077572199	0.2251	0.04057	<0.0001	1.252447955
Sex of child (ref = Male)								
Female	−0.08593	0.01579	<0.0001	0.917658465	−0.1305	0.02224	<0.0001	0.877656493
Education Level (ref = Secondary)								
Higher	−0.1301	0.03755	0.0005	0.878007626	−0.3136	0.04007	<0.0001	0.730811294
No education	0.5207	0.02411	<0.0001	1.683205481	0.2387	0.03452	<0.0001	1.2695976
Primary	0.4127	0.02551	<0.0001	1.51089169	0.1829	0.03626	<0.0001	1.200694333
Are there ways to prevent malaria (ref = Do not know)								
Yes	−0.1263	0.03226	<0.0001	0.881350402	–	–	–	–

The results in Table 3 indicate the parameter estimates, *p*-values, and odds ratio (OR). The current study reported only the independent variables with a statistically significant impact on anemia and malaria (*p*-value<0.05). The independent variables that have a statistically significant effect on both anemia and malaria are region, altitude, age of child in months, toilet facility of the household, main wall material used for the house, main roof material used for the house, children under five who slept under a mosquito net, whether the child had a fever in last 2 weeks before the survey took place, place of residence where the child resides, household wealth index, sex of the child, and mother’s education level. However, whether the mother knows of ways to prevent malaria or not did not have a statistically significant effect on anemia.

The results from Table 3 indicated that children from the central region of Nigeria were 0.064 (AOR = 0.064, *p*-value<0.0001) times less likely to test positive for malaria using RDT compared to children from the southern region. Similarly, children from the northern region of Nigeria were 0.082 (AOR = 0.082, *p*-value<0.0001) times less likely to test positive for malaria than their counterparts from the south. However, the prevalence of anemia showed an opposite trend. Children in the central region were about 1.873 (AOR = 1.873, *p*-value = 0.0003) times more likely to be anemic, while those in the northern region were 2.485 (AOR = 2.485, *p*-value<0.00001) times more likely to be anemic compared to children from the southern region. These results indicate that while malaria prevalence is lower in the central and northern regions, anemia is more prevalent.

Based on the results altitude was another key factor affecting anemia and malaria. Children living at lower altitudes (0–500 meters) were 1.246 (AOR = 1,246, *p*-value = 0.003) times more likely to test positive for malaria using RDT, and those at 501–1000 meters were 1.764 (AOR = 1.764, *p*-value<0.0001) times more likely test positive compared to children living at altitudes above 1,000 meters. Children from households located at 0–500 meters were 1.952 (AOR = 1.951, *p*-value<0.0001) times more likely to have anemia, while those at 501–1000 meters were 1.482 (AOR = 1.482, *p*-value<0.0001) times more likely to have anemia compared to children living at higher altitudes (greater than 1,000 meters). This finding suggest that low-altitude regions are particularly vulnerable to both anemia and malaria.

Children aged 12–24 months were 1.685 (AOR = 1.685, *p*-value<0.0001) times more likely to test positive for malaria compared to infants aged 6–12 months. The risk increased with age, with children aged 25–36 months, 37–48 months, and 49–59 months being 2.232 (AOR = 2.232, *p*-value<0.0001), 2.529 (AOR = 2.529, *p*-value<0.0001), and 3.200 (AOR = 3.199, *p*-value<0.0001) times more likely, respectively, to test positive. Conversely, anemia prevalence decreased as children grew older. Compared to infants aged 6–12 months, children aged 12–24 months, 25–36 months, 37–48 months, and 49–59 months being 0.728 (AOR = 0.728, *p*-value<0.0001), 0.447 (AOR = 0.447, *p*-value<0.0001), 0.335 (AOR = 0.335, *p*-value<0.0001), and 0.314 (AOR = 0.314, *p*-value<0.0001) times less likely, respectively, to have anemia. These results indicate that anemia is most prevalent among younger children, while older children face a greater risk of malaria.

The results in Table 3 show that children from households with different types of toilet facilities exhibited varying risks of malaria and anemia. Children from a households with latrine toilet facilities were 1.172 (AOR = 1.172, *p*-value<0.0001) times more likely to test positive for malaria using RDT compared to those from households with flush toilet. Similarly, children from households with no toilet facility were 1.333 (AOR = 1.333, *p*-value<0.0001) times more likely to test positive for malaria compared to those with flush toilets. Furthermore, children from households with latrine toilet facilities were 1.291 (AOR = 1.291, *p*-value<0.0001) times more likely to have anemia than those with flush toilets. Similarly, children from households with no toilet facility were 1.260 (AOR = 1.260, *p*-value<0.0001) times more likely to have anemia compared to children with flush toilets. These findings suggest that inadequate sanitation infrastructure is associated with increased risks of both anemia and malaria.

The study further revealed that children from households with walls made of bricks, cement, no walls, or other materials were 0.639 (AOR = 0.639, *p*-value<0.0001), 0.703 (AOR = 0.703, p-value<0.0001), 0.552 (AOR = 0.552, *p*-value<0.0001), and 0.526 (AOR = 0.526, *p*-value<0.0001) times less likely, respectively, to test positive for malaria using RDT compared to children from households with wood/mud walls. Similarly, children from households with walls made of bricks, cement, and no walls were 0.766 (AOR = 0.766, *p*-value = 0.0014), 0.782 (AOR = 0.782, *p*-value<0.0001), and 0.809 (AOR = 0.809, *p*-value = 0.003), times less likely, respectively, to have anemia compared to children from households with wood/mud walls. These findings indicate that durable wall materials, such as bricks or cement, and the absence of walls (allowing for better ventilation) may reduce the risk of both anemia and malaria.

Children from households with roof made of asbestos and metal/zinc were 1.258 (AOR = 1.258, *p*-value = 0.0037) and 1.165 (AOR = 1.165, *p*-value<0.0001) times more likely, respectively, to test positive for malaria compared to children with wooden roofs. In contrast, children from households with roofs made of other materials or thatch/palm leaves were 0.797 (AOR = 0.797, *p*-value = 0.0045) and 0.697 (AOR = 0.697, *p*-value<0.0001) times less likely, respectively, to test positive for malaria compared to children with wooden roofs. Children from households with roofs made of thatch/palm leaves were 0.774 (AOR = 0.774, *p*-value<0.0001) times less likely to have anemia than children from households with wooden roofs. The findings suggest that certain roof materials, particularly those that allow ventilation may be associated with reduced risks, while enclosed, heat-retaining materials may increase vulnerability to malaria.

Children from households where all children slept under a mosquito net were 0.886 (AOR = 0.886, *p*-value<0.0001) times less likely to test positive for malaria compared to those from households where only some children slept under a net. Similarly, children from households where none of the children slept under a mosquito net were 0.917 (AOR = 0.917, *p*-value = 0.0004) times less likely to test positive for malaria compared to children from households where some children slept under a net. Children from households where all children slept under a mosquito net were 0.920 (AOR = 0.920, *p*-value = 0.0235) times less likely to have anemia than those from households where some children slept under net. These results suggest that consistent and widespread use of mosquito nets within households provides a protective effect against both anemia and malaria.

Children who did not experience fever 2 weeks before the survey were 0.587 (AOR = 0.587, *p*-value<0.0001) times less likely to test positive for malaria than compared to those with a recent fever. For children whose mothers/caregivers were unaware of their fever status, the likelihood of testing positive for malaria were about 0.535 (AOR = 0.535, *p*-value<0.0001) times less likely to test positive for malaria than children with a recent fever. Children without a recent fever were 0.682 (AOR = 0.682, *p*-value<0.0001) times less likely to have anemia, while those whose mothers/caregivers were unaware of their fever status were 0.419 (AOR = 0.419, *p*-value<0.0001) times less likely to have anemia compared to children with a recent fever. These findings emphasize the link between febrile illness and increased risks of both anemia and malaria.

Children from rural households were 1.312 (AOR = 1.312, *p*-value<0.0001) times more likely to test positive for malaria compared to those from urban households. Similarly, children from rural households were 1.132 (AOR = 1.132, *p*-value<0.0001) times more likely to have anemia than their urban counterparts. This rural–urban divide highlights the greater burden of disease in rural areas.

Children from the poorest households were 3.198 (AOR = 3.198, *p*-value<0.0001) times more likely to test positive for malaria compared to children from the richest wealth class. Similarly, children from poorer, middle, and richer households were 2.751 (AOR = 2.751, *p*-value<0.0001), 2.185 (AOR = 2.185, *p*-value<0.0001), and 2.078 (AOR = 2.078, *p*-value<0.0001) times more likely, respectively, to test positive for malaria. Children for the poorest households were 1.700 (AOR = 1.699, *p*-value<0.0001) times more likely to have anemia compared to children from the richest wealth class. Children from poorer, middle, and richer households were 1.470 (AOR = 1.470, *p*-value<0.0001), 1.279 (AOR = 1.279, *p*-value<0.0001), and 1.253 (AOR = 1.253, *p*-value<0.0001) times more likely, respectively, to have anemia than those from the richest wealth class. These results underscore the disproportionate burden of anemia and malaria among children from poor households. Female children were 0.918 (AOR = 0.918, *p*-value<0.0001) times less likely to test positive for malaria compared to male children. Similarly, female children were 0.878 (AOR = 0.878, *p*-value<0.0001) times less likely to have anemia than male children. This suggests that male children may face a slightly higher risk of both conditions compared to female children.

Children whose mothers/caregivers had primary education, or no education were 1.511 (AOR = 1.511, *p*-value<0.0001) and 1.683 (AOR = 1.683, *p*-value<0.0001) times more likely, respectively, to test positive for malaria compared to children whose mothers/caregivers had secondary education. Conversely, children whose mothers had higher education were 0.878 (AOR = 0.878, *p*-value = 0.0005) times less likely to test positive for malaria than those whose mothers/caregivers had secondary education. Similarly, children whose mothers/caregivers had had primary education, or no education were 1.201 (AOR = 1.201, *p*-value<0.0001) and 1.270 (AOR = 1.270, *p*-value<0.0001) times more likely, respectively, to have anemia compared to children whose mothers/caregivers had secondary education. Children whose mothers/caregivers had higher education were 0.731 (AOR = 0.731, *p*-value<0.0001) times less likely to have anemia than those whose mothers/caregivers had secondary education. These findings highlight the protective effect of maternal education, particularly higher education, against both anemia and malaria.

Maternal knowledge of malaria prevention also influenced malaria prevalence. Children whose mothers/caregivers were knowledgeable about ways to prevent malaria were 0.881 (AOR = 0.881, *p*-value<0.0001) times less likely to test positive for malaria compared to those whose mothers/caregivers’ lacked knowledge about preventative measures. This suggests that awareness and understanding of malaria prevention among mothers/caregivers play a crucial role in reducing the risk of the disease in children.

The independent variables that have a statistically significant effect on both anemia and malaria are region, altitude, age of the child in months, toilet facility of the household, main wall material used for the house, main roof material used for the house, children under five who slept under a mosquito net, whether the child had a fever in last 2 weeks before the survey took place, place of residence where the child resides, household wealth index, sex of the child, and mother’s education level. However, whether the mother knows of ways to prevent malaria or not did not have a statistically significant effect on anemia. The study also investigated the interaction effects. However, none of them were statistically significant. Therefore, they were not included in the analysis.

## Discussion

In the current study, the power was calculated to check if the selected sample will validate the ability to detect true associations. The value obtained for the power (Power > 0.999) showed that the sample selected was sufficient, which indicated that the study has a high probability of detecting a statistically significant effect, and that the parameter estimates found in the model are accurate. The advantage of the joint model is that it allows anemia and malaria to be analyzed simultaneously, capturing their potential relationship and shared influencing factors (13, 25). In the current study, the joint generalized linear mixed model was employed to determine anemia and malaria among children between 6 and 59 months old in Nigeria. The 2021 Nigeria Indicator Malaria Indicator (NIMS) data create the sample. The joint generalized linear mixed model results were obtained from SAS online University Edition. The results from the covariance test showed that there is a strong correlation between malaria and anemia status. There are different studies in the literature that have used the joint model (13, 25, 26); however, in this study, the missing values were catered for by using multiple imputations by chained equations also known as the fully conditional specification (FCS) (15). The estimates obtained from the complete case analysis and the imputed data were compared and, based on the standard errors of the imputed data, were lower than those from the complete case; this implied that the imputed data proved to have reliability when it comes to the outcome of the model (15). The key factors obtained from the study are divided into the following sections, which are environmental, health-related, and socioeconomic.

### Environmental factors

The altitude of the household also contributed significantly to malaria and anemia; based on the study’s outcome, children from households at an altitude greater than 1,000 meters are less likely to test positive for malaria or have anemia. This could result from mosquitoes developing better in hot areas, which can be experienced in areas with low altitudes (9, 27). The study revealed that children who slept under a mosquito net the night before the survey had a lower risk of having malaria and anemia. This might be because children who sleep under mosquito bed nets are being protected from mosquito bites. The result is consistent with what has been found in previous studies, such as those by Gaston and Ramroop (9). The study by Gayawan et al. (5) checked whether a household has a net; however, in this study, the usage of mosquito nets was checked and found to be statistically significant for malaria and anemia.

### Health-related factors

The current study shows that malaria increases as the child grows, and anemia decreases for the same group of children (13). Children whose mother did not know their fever status in the past 2 weeks before the survey was conducted and those who did not have a fever in the past 2 weeks were at low risk of testing positive for malaria and having anemia. Those who had a fever in the past 2 weeks before the survey was conducted were at a high risk of testing positive for malaria using RDT and having anemia. This suggests that mothers/caregivers of children under 5 years should be educated about fever and how this might be one of the symptoms of malaria infection. Female children are less likely to test positive for malaria using RDT and to have anemia compared to male children. Moreover, children whose mothers knew of ways to prevent malaria were less likely to test positive for malaria using RDT compared to those who did not know of ways to prevent malaria.

### Socioeconomic factors

The children from the central and north regions show a decline in malaria compared to those from the south region; however, the opposite is true for anemia. Therefore, this suggests that if the environment is controlled for malaria, an effective reduction in anemia will be observed (28, 29). Additionally, if anemia is controlled in an area with a high prevalence of malaria, it can effectively reduce childhood mortality related to malaria (30). The study further showed that proper sanitation would significantly reduce malaria and anemia in children from Nigeria. Children from a household where the main toilet facility is a latrine, or there is no facility at all are at a high risk of contracting malaria and having anemia. The main wall material used for the construction of the house also plays a significant role in malaria and anemia; based on the results, kids who are from a household where the main wall material is wood/mud are at a high risk of testing positive for malaria using RDT and having anemia. The study further showed that children from households with the main roof material asbestos and metal/zinc are at a higher risk of testing positive for malaria using RDT than those with wood as the main roof material. Additionally, children who are from a household where the main roof material is other and thatch/palm leaf are at low risk of testing positive for malaria using RDT compared to those from a household with wood as the main roof material. However, children from households with the main roof material thatch/palm leaf are less likely to have anemia than those from households with wood. This is the only category that was statistically significant for anemia.

The type of place of residence showed a significant effect on malaria and anemia, where children from rural areas are at a high risk of testing positive for malaria and having anemia, compared to those who are from urban areas of Nigeria. Previous studies focusing on malaria and anemia as separate response variables have established the links between the type of residence and the response variables (9, 26, 31). One of the possible reasons for this outcome is that rural dwellers are often ignorant about malaria prevention strategies and, as a result, are unable to know about the available programs being offered to benefit them (5, 32).

As the household wealth index increases from poorest to richest, the chance of children from those households testing positive for malaria using RDT decreases, the same as having anemia. The emphasis on education should not be taken for granted in countries where there is a high prevalence of malaria or anemia. Based on the current study, it has been noted that as the mother’s education level increases, the chances of testing positive for malaria or having anemia decrease. Similar results were observed in the literature (4, 9, 13, 25, 26, 30).

In the current study, there was an indication that malaria and anemia are strongly associated; this means that when there is an increase in malaria in children, there is an increase in anemia. This result is consistent with what has been observed in a study by other studies in the literature (13, 33). The inverse is also true; when malaria reduces in children, so is anemia.

## Conclusion

This study aimed to determine the risk factors associated with positive malaria RDT results and anemia. The current study used the joint generalized linear mixed model. The statistical model used to estimate the risk factors associated with a positive RDT result and anemia in children under 5 years of age belongs to the family of generalized linear models (GLM). The generalized linear mixed models (GLMMs) effectively consider the fixed effects. Furthermore, the assumption of normality is relaxed (34), which is an advantage for using the method in a survey dataset. In this study, the missing observations were included; the multiple imputation using chained equations (MICE), also known as FCS in SAS, was used to address the missingness of the observations. Thus, modeling the complete and advantageous dataset results in a more accurate result.

The current study revealed that there is an association between socioeconomic, demographic, and geographical factors with malaria and anemia in children under 5 years in Nigeria. The independent variables that have a statistically significant effect on both anemia and malaria are region, altitude, age of the child in months, toilet facility of the household, main wall material used for the house, main roof material used for the house, children under five who slept under a mosquito net, whether the child had a fever in last 2 weeks before the survey took place, place of residence where the child resides, household wealth index, sex of the child, and mother’s education level. However, whether the mother knows of ways to prevent malaria or not did not have a statistically significant effect on anemia.

These factors can be used to identify hotspots of malaria and anemia, thus, in turn, allowing preventative measures to be put in place to curb the increase in malaria anemia cases in children. The government should focus on the more disadvantaged households from rural areas with low altitude levels, especially those with poor toilet facilities. The findings also reveal that mothers’ education should be encouraged and supported. The current study will help the government and policymakers to control and possibly eradicate malaria and anemia in children under 5 years of age in Nigeria. The approach can be to educate caregivers on malaria risk factors and some of the available ways to prevent malaria. Furthermore, awareness campaigns can be started through the radio or the media platforms available to the wider community. The primary focus should be on children who are from households which are located at an altitude that is less than 1,000 m, children who had a fever in the last 2 weeks before the survey was conducted, those with poor toilet facilities and poor housing, those from the rural areas, and lastly male children.

According to the NIMS 2021 report, it has been outlined that about 24% of mosquito nets were not used the night before the survey. The main reasons given for not using a mosquito net the night before the survey were that net was not needed (24%), there were no mosquitoes (18%), it was too hot (16%), and other (12%). The proportion of respondents reporting that they did not use a net the night before the survey because it was not needed was higher in rural (26%) than urban (20%) areas. Taking into consideration the statistics outlined above, it is suggested that there is a lack of knowledge on why and when mosquito nets should be used. The data collection took place from October to December, which is deemed as summer in the African continent, and therefore there is a high prevalence of mosquitoes.

Based on the current study’s findings, which shows that the use of mosquito nets is vital to fight against the high prevalence of malaria in children under 5 years. It is therefore suggested that local governments and health organizations should engage community leaders from both rural and urban areas to work together to develop strategies to combat malaria, through the education of why and when mosquito nets should be used. This can be achieved through the rolling out of mosquito nets and giving proper training on how to use them for effective protection.

The policymakers and the Nigerian government should focus on improving the housing facilities, giving more attention to children with fever, and providing more mosquito nets to households in rural areas that are at an altitude of less than 1,000 meters.

### Future studies

Longitudinal studies can be used to address the problem of causality in future studies. Even though each category has significant risk factors: demographic, economic, and geographical. It might be worthwhile for future studies to investigate the interactions between some of the predictor variables to understand how they contribute to malaria and anemia prevalence. Other studies can also investigate plans where there are going to be new developments and advice accordingly based on some of the risk factors that have been outlined in the current study to make sure that, for instance, the altitude level is high enough to avoid the high prevalence of malaria and anemia in children. Furthermore, the contributing factors to anemia can be added to determine which ones add more significance to positive RDT results.

### Limitations

This study used secondary cross-sectional data from NMIS, and this data may not be able to address the causality but rather the association. Therefore, a longitudinal study is suggested to address this problem.

## Data Availability

Publicly available datasets were analyzed in this study. This data can be found at: https://dhsprogram.com/data/.

## References

[1] Organization WH, others. World malaria report. Geneva: World Health Organization (2022, 2022).

[2] Molla A Egata G Mesfin F Arega M Getacher L. Prevalence of Anemia and associated factors among infants and young children aged 6–23 months in Debre Berhan town, North Shewa, Ethiopia. Huerta JM, editor. J Nutr Metab. (2020) 2020:1–12. doi: 10.1155/2020/2956129, PMID: 33414958 PMC7768586

[3] Menon MP Yoon SS. Prevalence and factors associated with Anemia among children under 5 years of age—Uganda, 2009. Am Soc Trop Med Hyg. (2015) 93:521–6. doi: 10.4269/ajtmh.15-0102, PMID: 26055748 PMC4559690

[4] Roberts D Matthews G. Risk factors of malaria in children under the age of five years old in Uganda. Malar J. (2016) 15:246. doi: 10.1186/s12936-016-1290-x, PMID: 27121122 PMC4848810

[5] Gayawan E Egbon OA Adebayo SB. Spatial modelling of the joint burden of malaria and anaemia co-morbidity in children: a Bayesian geoadditive perspective. Commun Stat Case Stud Data Anal Appl. (2022) 8:264–81. doi: 10.1080/23737484.2022.2031345, PMID: 39613955

[6] Wanzira H Katamba H Okullo AE Agaba B Kasule M Rubahika D. Factors associated with malaria parasitaemia among children under 5 years in Uganda: a secondary data analysis of the 2014 malaria Indicator survey dataset. Malar J. (2017) 16:191. doi: 10.1186/s12936-017-1847-3, PMID: 28482832 PMC5423009

[7] National Malaria Elimination Programme (NMEP) [Nigeria], National Population Commission (NPC) [Nigeria], and ICF. Nigeria malaria Indicator survey 2021 final report. (2022) Available at: https://dhsprogram.com/pubs/pdf/MIS41/MIS41.pdf

[8] Egbon O. Bayesian analysis of the joint risk of malaria and Anemia among under-age Ve children in Nigeria. (2021). Available at: https://www.researchsquare.com/article/rs-148795/v1

[9] Gaston RT Ramroop S. Prevalence of and factors associated with malaria in children under five years of age in Malawi, using malaria indicator survey data. Heliyon. (2020) 6:e03946. doi: 10.1016/j.heliyon.2020.e03946, PMID: 32426545 PMC7226652

[10] Zahouli JZB Edi CAV Yao LA Lisro EG Adou M Koné I. Small-scale field evaluation of PermaNet® dual (a long-lasting net coated with a mixture of chlorfenapyr and deltamethrin) against pyrethroid-resistant *Anopheles gambiae* mosquitoes from Tiassalé, Côte d’Ivoire. Malar J. (2023) 22:36. doi: 10.1186/s12936-023-04455-z, PMID: 36726160 PMC9893697

[11] Zewude BT Debusho LK. Prevalence rate and associated risk factors of Anaemia among under five years children in Ethiopia. Nutrients. (2022) 14:2693. doi: 10.3390/nu14132693, PMID: 35807875 PMC9268795

[12] Arshad M Jaleel H Iqbal S Asif M Ali M Mubarak M. Comorbidities affect the recovery rate of Covid-19 patients - a retrospective study in Lahore, Pakistan. Med Res Arch [Internet]. (2022) 10. doi: 10.18103/mra.v10i9.3060

[13] Gaston RT Ramroop S Habyarimana F. Joint modelling of malaria and anaemia in children less than five years of age in Malawi. Heliyon. (2021) 7:e06899. doi: 10.1016/j.heliyon.2021.e06899, PMID: 34027150 PMC8121655

[14] Finkle WD Greenland S Miettinen OS Ziel HK. Endometrial cancer risk after discontinuing use of unopposed conjugated estrogens (California, United States). Cancer Causes Control. (1995) 6:99–102. doi: 10.1007/BF00052769, PMID: 7749058

[15] Hendry GM Naidoo RN Zewotir T North D Mentz G. Model development including interactions with multiple imputed data. BMC Med Res Methodol. (2014) 14:136. doi: 10.1186/1471-2288-14-136, PMID: 25524532 PMC4289583

[16] Klebanoff MA Cole SR. Use of multiple imputation in the epidemiologic literature. Am J Epidemiol. (2008) 168:355–7. doi: 10.1093/aje/kwn071, PMID: 18591202 PMC2561989

[17] Mirzaei A Carter SR Patanwala AE Schneider CR. Missing data in surveys: key concepts, approaches, and applications. Res Soc Adm Pharm. (2022) 18:2308–16. doi: 10.1016/j.sapharm.2021.03.009, PMID: 33775556

[18] Stavseth MR Clausen T Røislien J. How handling missing data may impact conclusions: a comparison of six different imputation methods for categorical questionnaire data. SAGE Open Med. (2019) 7:205031211882291. doi: 10.1177/2050312118822912, PMID: 30671242 PMC6329020

[19] Rubin DB. The calculation of posterior distributions by data augmentation: comment: a noniterative sampling/importance resampling alternative to the data augmentation algorithm for creating a few imputations when fractions of missing information are modest: the SIR algorithm. J Am Stat Assoc. (1987) 82:543. doi: 10.2307/2289460

[20] Lee KJ Carlin JB. Multiple imputation for missing data: fully conditional specification versus multivariate Normal imputation. Am J Epidemiol. (2010) 171:624–32. doi: 10.1093/aje/kwp425, PMID: 20106935

[21] World malaria report 2022. Geneva: World Health Organization (2022).

[22] Kamya MR Arinaitwe E Wanzira H Katureebe A Barusya C Kigozi SP. Malaria transmission, infection, and disease at three sites with varied transmission intensity in Uganda: implications for malaria control. Am Soc Trop Med Hyg. (2015) 92:903–12. doi: 10.4269/ajtmh.14-0312, PMID: 25778501 PMC4426576

[23] Kiggundu VL O’Meara WP Musoke R Nalugoda FK Kigozi G Baghendaghe E. High prevalence of malaria Parasitemia and Anemia among hospitalized children in Rakai, Uganda. PLoS One. (2013) 8:e82455. doi: 10.1371/journal.pone.0082455 24358185 PMC3866122

[24] Olukosi AY Agomo CO Aina OO Akindele SK Okoh HO Brai BC. Prevalence of malaria and anaemia during the dry season in North Central and South Western Nigeria. J Parasitol Vector Biol. (2018).

[25] Habyarimana F. Key determinants of malnutrition of children under five years of age in Rwanda: simultaneous measurement of three anthropometric indices. (2016) Available at: http://aps.journals.ac.za/pub/article/view/836

[26] Khulu C Ramroop S Habyarimana F. Modelling factors associated with malnutrition and Anemia in children under five years in Angola, Senegal, and Malawi by using a joint model. Open Public Health J. (2023) 16:e187494452212164. doi: 10.2174/18749445-v15-e221220-2022-82

[27] Chirombo J Lowe R Kazembe L. Using structured additive regression models to estimate risk factors of malaria: analysis of 2010 Malawi malaria Indicator survey data. PLoS One. (2014) 9:e101116. 24991915 10.1371/journal.pone.0101116 PMC4084636

[28] Hershey CL Florey LS Ali D Bennett A Luhanga M Mathanga DP. Malaria control interventions contributed to declines in malaria Parasitemia, severe Anemia, and all-cause mortality in children less than 5 years of age in Malawi, 2000–2010. Am J Trop Med Hyg. (2017) 97:76–88. doi: 10.4269/ajtmh.17-0203, PMID: 28990920 PMC5619935

[29] Yimgang DP Buchwald AG Coalson JE Walldorf JA Bauleni A Kapito-Tembo A. Population attributable fraction of Anemia associated with plasmodium falciparum infection in children in southern Malawi. Am J Trop Med Hyg. (2021) 104:1013–7. doi: 10.4269/ajtmh.20-1120, PMID: 33399043 PMC7941802

[30] Seyoum S. Analysis of prevalence of malaria and Anemia using bivariate Probit model. Ann Data Sci. (2018) 5:301–12. doi: 10.1007/s40745-018-0138-3

[31] Gayawan E Arogundade ED Adebayo SB. Possible determinants and spatial patterns of anaemia among young children in Nigeria: a Bayesian semi-parametric modelling. Int Health. (2014) 6:35–45. doi: 10.1093/inthealth/iht034, PMID: 24486460

[32] Abimbola S Olanipekun T Schaaf M Negin J Jan S Martiniuk ALC. Where there is no policy: governing the posting and transfer of primary health care workers in Nigeria. Int J Health Plann Manag. (2017) 32:492–508. doi: 10.1002/hpm.2356, PMID: 27144643 PMC5716250

[33] Noland GS Ayodo G Abuya J Hodges JS Rolfes MAR John CC. Decreased prevalence of Anemia in Highland areas of low malaria transmission after a 1-year interruption of transmission. Clin Infect Dis. (2012) 54:178–84. doi: 10.1093/cid/cir768, PMID: 22052892 PMC3245725

[34] Lin X Zhang D. Inference in generalized additive mixed models by using smoothing splines. J R Stat Soc Ser B Stat Methodol. (1999) 61:381–400. doi: 10.1111/1467-9868.00183

